# Genome-wide identification and systematic analysis of the *HD-Zip* gene family and its roles in response to pH in *Panax ginseng* Meyer

**DOI:** 10.1186/s12870-023-04038-9

**Published:** 2023-01-13

**Authors:** Li Li, Boxin Lv, Kaiyou Zang, Yue Jiang, Chaofan Wang, Yanfang Wang, Kangyu Wang, Mingzhu Zhao, Ping Chen, Jun Lei, Yi Wang, Meiping Zhang

**Affiliations:** 1 College of Life Science, Jilin Agricultural University, Changchun, Jilin, 130118 China; 2 Research Center for Ginseng Genetic Resources Development and Utilization, Jilin Province, Jilin Agricultural University, Changchun, Jilin, 130118 China; 3 College of Chinese Medicinal Materials, Jilin Agricultural University, Changchun, Jilin, 130118 China

**Keywords:** *PgHDZ* gene family, Phylogeny, Diversity and evolution, Expression pattern, Co-expression network, Ginseng

## Abstract

**Background:**

Ginseng, *Panax ginseng* Meyer, is a traditional herb that is immensely valuable both for human health and medicine and for medicinal plant research. The *homeodomain leucine zipper* (*HD-Zip*) gene family is a plant-specific transcription factor gene family indispensable in the regulation of plant growth and development and plant response to environmental stresses.

**Results:**

We identified 117 *HD-Zip* transcripts from the transcriptome of ginseng cv. Damaya that is widely grown in Jilin, China where approximately 60% of the world’s ginseng is produced. These transcripts were positioned to 64 loci in the ginseng genome and the ginseng *HD-Zip* genes were designated as *PgHDZ* genes. Identification of 82 and 83 *PgHDZ* genes from the ginseng acc. IR826 and cv. ChP genomes, respectively, indicated that the *PgHDZ* gene family consists of approximately 80 *PgHDZ* genes. Phylogenetic analysis showed that the gene family originated after Angiosperm split from Gymnosperm and before Dicots split from Monocots. The gene family was classified into four subfamilies and has dramatically diverged not only in gene structure and functionality but also in expression characteristics. Nevertheless, co-expression network analysis showed that the activities of the genes in the family remain significantly correlated, suggesting their functional correlation. Five hub *PgHDZ* genes were identified that might have central functions in ginseng biological processes and four of them were shown to be actively involved in plant response to environmental pH stress in ginseng.

**Conclusions:**

The *PgHDZ* gene family was identified from ginseng and analyzed systematically. Five potential hub genes were identified and four of them were shown to be involved in ginseng response to environmental pH stress. The results provide new insights into the characteristics, diversity, evolution, and functionality of the *PgHDZ* gene family in ginseng and lay a foundation for comprehensive research of the gene family in plants.

**Supplementary Information:**

The online version contains supplementary material available at 10.1186/s12870-023-04038-9.

## Background

Ginseng, *Panax ginseng* Meyer, is a traditional herb that belongs to the *Araliaceae* family [1]. The dry roots of ginseng have been used as a precious herb in human medicine for thousands of years and shown recently to have remarkable therapeutic functions for several diseases, such as cancer [2], obesity [3], cardiovascular disorders [4], and neurological disorders [5]. Furthermore, ginseng also serves as a functional ingredient for many healthcare products, functional foods, and cosmetics [6]. A relic from the Tertiary era [7] indicated that some plant growth and development characteristics of ginseng had limited its planting and production, such as long generation period, slow growth rate [1], and sensitivity to environmental conditions [8]. It is also essential to comprehensively understand the complexity of ginseng leaf change and secondary metabolite biosynthesis as its age increases. Therefore, it is vital to both ginseng research and application to analyze the characteristics and formation mechanism of its plant.

Transcription factors (TFs) are DNA-binding proteins that regulate the transcription of DNA to mRNA via binding *cis*-regulatory elements [9]. In plants, approximately 10% of their genes encode TFs [10] that have a wide range of functions in plant growth [11], development [12], stress response [13], and secondary metabolite biosynthesis [14]. In ginseng, 3588 [1] and 2556 [15] TFs have been identified from the genomes of different varieties. Ginseng TF gene families, *bHLH* [16, 17], *GRAS* [18], *WRKY* [19], *SPL* [20], *NAC* [21], *AP2/ERF* [22], and *MYB* [23], have been identified and systematically characterized. The vital roles of several ginseng TF genes have been verified, including responsiveness to brassinosteroid during storage root formation [24], responsiveness to salt and hormones [25], and biosynthesis of ginsenosides [26]. These studies have laid a foundation for deciphering the molecular mechanisms underpinning ginseng’s biological characteristics, growth, and development.

The *homeodomain leucine zipper* (*HD-Zip*) gene family is a unique TF family in plants that is characterized by a highly conserved homeodomain (HD) and a homeobox-associated leucine zipper domain (HALZ) closely linked to its carboxy-terminal region (CTR) [27]. As the basic domain of the HD superfamily, the HD domain is a conserved residue of approximately 60 amino acids that fold into a three-helix DNA-interacting structure and carry out the function of DNA-binding [28]. The HALZ domain is less conserved in the *HD-Zip* genes and acts as a dimerization motif for DNA binding [29]. Furthermore, the *HD-Zip* gene family is classified into four subfamilies, defined as subfamily I through IV, according to the existence of additional conserved domains or motifs [29]. Subfamily I usually only has the basic HD and HALZ domains, but the existence of other conserved motifs may explain their functional divergence, such as the transactivation AHA motifs at the CTR [30]. Subfamily II is characterized by the presence of two exclusive motifs besides the basic HD and HALZ domains: the CPSCE motif in the CTR and the ZIBEL-like motif at the amino-terminal region (NTR) [28]. Both subfamily III and subfamily IV have the START (STeroidogenic Acute Regulatory protein-related lipid Transfer) domain following the basic HD domain, but subfamily III is characterized by additional presence of a Per-ARNT-Sim-like (PAS-like) MEKHLA domain at the CTR [31]. Evolutionary analysis showed that the *HD-Zip* gene family appeared during early chlorophyte evolution, diverged into the four subfamilies during early charophyte evolution, and experienced multiple duplication events in land plants [28].

The *HD-Zip* gene family plays important roles in plant growth and development, such as embryogenesis [32] and the formation and development of various tissues [33]. The genes in the same subfamily tend to have the related functions or to be involved in the same biological process. For example, the subfamily II *HD-Zip* genes are mainly involved in the organogenesis and regulation related to the photosynthetic process [34, 35]. The subfamily III *HD-Zip* genes are mainly involved in the vascular occurrence and polarity regulation of plant leaves and stems [36]. The subfamily I *HD-Zip* genes are widely involved in plant response to abiotic stresses [37] and the subfamily IV *HD-Zip* genes are involved in plant protection from biotic and abiotic stresses by regulating the differentiation and maintenance of outer cell layers and biosynthesis and transport of lipid [38]. In particular, these functions of *HD-Zip* genes are widely related to plant hormone regulation, such as abscisic acid, gibberellin, ethylene, and brassinosteroid [39]. The feedback regulation mechanism formed between *HD-Zip* genes or with other related genes is of great significance in the performance of gene function [40]. Moreover, several *HD-Zip* genes have been reported to have regulatory functions in the biosynthesis of secondary metabolites including triterpene [41], flavanol [42], and catechin [43]. However, the *HD-Zip* gene family in ginseng has not been reported yet.

Suitable pH in soil is essential for plant growth and development, but the pH values of soil frequently change worldwide as fertilizer applications and agricultural irrigation increase. The increase or decrease of soil pH will lead to a decrease in ecosystem biodiversity, the reduction of microbial activities, and thus a decline in crop productivity [44, 45]. Therefore, it would be beneficial to crop production to study the regulation pathway of plant response to pH change, thereby increasing plant tolerance to pH variation. Several TF gene families have been reported to have regulatory functions in plant response to the pH change, such as *WRKY* [46], *bHLH*, *C2H2*, and *bZIP* [47], but the *HD-Zip* gene family has not been reported to be involved in plant response to pH change yet.

In the present study, we identified the *HD-Zip* gene family (hereafter defined *PgHDZ* gene family) from ginseng, including the ginseng cv. Damaya transcriptome [48] and the ginseng acc. IR826 [15] and cv. ChP genomes [1], and systematically analyzed through conserved motif prediction, gene structure characterization, phylogenetic analysis, gene ontology (GO) categorization, gene expression patterning, and co-expression network analysis. Furthermore, we identified five hub genes from the *PgHDZ* family and verified their functions in plant responses to pH change in ginseng. The findings of this study provide comprehensive insights into the characterization of the *PgHDZ* gene family and its functionality in plant responses to pH variation and lay a foundation for in-depth study of its function in ginseng and other plant species.

## Methods

### Databases

The datasets analyzed during the current study are available in the *Panax ginseng* 14 tissues transcriptomes repository (https://www.ncbi.nlm.nih.gov/bioproject/?term=PRJNA302556) and Gene Expression Omnibus of NCBI (SRP066368 and SRR13131364 – SRR13131405). The transcriptome of ginseng cv. Damaya previously developed [48] was used for *PgHDZ* gene identification and expression characterization. Moreover, two other related databases of gene expressions were also used for this study, with one developed from the roots of 5-, 12-, 18-, and 25-year-old ginseng cv. Damaya plants and the other from the four-year-old ginseng roots of 42 representative cultivars collected from Jilin, China. Finally, the ginseng cv. ChP [1] and acc. IR826 [15] draft genomes and the ginseng chromosome-sized genome [49] were used for the *PgHDZ* gene identification and the genome localization, respectively.

### Identification of the *PgHDZ* gene family and its subfamilies

The basic conserved domains of the plant *HD-Zip* gene family, the HD and HALZ domains, have been widely used for identification of the *HD-Zip* gene family in plant species. However, Schrick et al. [50] reported that some genes of the *HD-Zip* subfamilies III and IV only have ZLZ (leucine zipper-loop-zipper) region, instead of HALZ. Therefore, some *HD-Zip* genes would be missed by simply using HD and HALZ domains for *HD-Zip* gene identification. Because genes with the ZLZ region definitely have the HD and START domains, the HD domain (PF00046), the HALZ domain (PF02183), and the START domain (PF01852) were used to identify the *HD-Zip* genes in ginseng. Therefore, the genes containing both HD and HALZ domains, or both HD and START domains would be identified as *HD-Zip* genes.

Three methods were used to ensure that the screening of the ginseng *HD-Zip* gene family was comprehensive. The first method was to download the conserved protein sequences of the HD, HALZ, and START domains of the plant *HD-Zip* genes from the PFAM database (https://pfam.xfam.org), then tblastn them against the ginseng transcriptome (*E*-value: 1.0E-05). The second method was to download all *HD-Zip* genes that have been reported in different species from the NCBI nucleotide database (https://www.ncbi.nlm.nih.gov), then use them as queries to blast against the ginseng transcriptome (*E*-value: 1.0E-05). The third method was to download the Hidden Markov Model of the HD, HALZ, and START domains of the plant *HD-Zip* genes from the PFAM database as queries to search the protein databases of the ginseng acc. IR826 and cv. ChP draft genomes, respectively, using HMMER software (version 3.0). Then, the ginseng proteins obtained from each genome were used as queries to blast against ginseng transcriptome (*E*-value: 1.0E-05). All sequences of the putative ginseng *HD-Zip* genes obtained above were confirmed by Conserve Domain Search of NCBI (https://www.ncbi.nlm.nih.gov/Structure/cdd/wrpsb.cgi) and the *HD-Zip* genes without the HD and HALZ/START domains were deleted. The confirmed *HD-Zip* genes were selected and defined as *PgHDZ* genes.

Furthermore, *PgHDZ* genes were clustered into four subfamilies by their conserved domains and motifs. Subfamily I contains the conserved domains, HD and HALZ. Subfamily II contains the conserved domains, HD, HALZ, and the conserved motif, CPSCE. Subfamily III contains the conserved domains, HD and START, and the conserved motif, MEKHLA. Subfamily IV contains the conserved domains, HD and START.

### The size of the *PgHDZ* gene family and its positions in the ginseng genome

To estimate the size of the *PgHDZ* gene family, the *PgHDZ* genes in IR826 [15] and ChP [1] genomes were first identified, respectively, using the above method. These *PgHDZ* genes were then positioned onto the ginseng 24 chromosomes [49] using the BLASTn method with criteria of query cover ≥ 180 bp (because the HD domain has approximately 60 amino acids in length), identity ≥ 99%, and *E*-value ≤ 1.0E-06. The distribution of the genes in the ginseng genome was visualized by the TBtools software (version 1.09876) [51].

### Motif prediction and structure analysis of the *PgHDZ* gene transcripts

The conserved domains and conserved motifs of the *PgHDZ* gene transcripts were identified by Conserve Domain Search of NCBI and MEME online tools with default parameters (http://meme-suite.org/tools/meme), respectively. The structures of the genes were shown by TBtools (version 0.64) using the phylogenetic trees constructed by MEGA (version 7.0.26) as the gene grouping criteria.

### Phylogenetic analysis of the *PgHDZ* gene family and estimation of Ka/Ks ratio in gene duplication

To decipher the phylogeny and evolution of the *PgHDZ* family, two phylogenetic trees were constructed for the gene family by MEGA. One was constructed only for the *PgHDZ* gene transcripts and the other constructed for the *HD-Zip* gene transcripts using the *HD-Zip* genes from several species as outgroups. These outgroup species were chosen based on their evolutionary tree previously reported, including mosses, gymnosperm, monocots, and eudicots (Table S1) so that the origin and evolution of the *PgHDZ* gene family could be inferred. The phylogenetic trees were constructed using the maximum likelihood method with the substitution model of the Tamura 3-parameter and the bootstraps were set to 2000. The Evolview online tool (http://www.evolgenius.info/evolview/) was used to prettify the phylogenetic trees.

The duplicated gene pairs of the *PgHDZ* gene family were identified by the alignment of their coding sequences via the Vector NTI software [52]. Gene pairs that had the highest identity were used to calculate the synonymous (Ka) and non-synonymous (Ks) substitution values, and the Ka/Ks ratio was estimated by utilizing the Ka/Ks Calculator online tool (http://services.cbu.uib.no/tools/kaks/). The time of gene duplication and divergence (million years ago) were estimated by a synonymous mutation rate of λ substitutions per synonymous site per year as T = Ks/2λ × 10^− 6^, where λ = 6.5 × 10^− 9^ [53].

### Annotation and gene ontology (GO) categorization of the *PgHDZ* gene transcripts

The functional differentiation of the *PgHDZ* gene transcripts was determined by gene annotation, followed by GO categorization using Blast2GO with default parameters. The numbers of the *PgHDZ* gene transcripts annotated and categorized into the Biological Process, Molecular Function, and Cellular Component categories and their subcategories (Level 2) were recorded and analyzed. The functional differentiation of transcripts that were clustered into different subfamilies was presented as a Venn diagram.

### Spatiotemporal expressions of the *PgHDZ* gene transcripts and their expression diversity among cultivars

The expressions of the *PgHDZ* gene transcripts were extracted from the database of (a) four aged plant roots, (b) 14 tissues of a four-year-old plant, and (c) four-year-old plant roots of 42 cultivars. The numbers of the *PgHDZ* gene transcripts expressed in different aged plant roots, different tissues of a four-year-old plant, and in the four-year-old plant roots of different cultivars were calculated and compared with the same number of unknown gene transcripts randomly selected from the ginseng transcriptome as the negative control to determine the expression patterns and diversity of the *PgHDZ* gene family. Furthermore, the expression heatmaps of these *PgHDZ* gene transcripts were conducted using R programming language with the heatmap package to present the spatiotemporal expression patterns of the *PgHDZ* gene transcripts and their diversity among cultivars.

### Co-expression networks of the *PgHDZ* genes transcripts and the potential hub *PgHDZ* genes

To construct the co-expression network of the *PgHDZ* gene family, the Spearman’s correlation coefficients of *PgHDZ* gene transcripts expressed in 14 tissues of a four-year-old ginseng plant and in four-year-old plant roots of 42 cultivars were calculated using the R language. The co-expression networks were visualized by BioLayout *Express*^3D^ software [54]. To reveal the characteristics of the network, the co-expression networks of the *PgHDZ* genes were constructed with *p*-values varying from 5.0E-02 to 1.0E-08, and the number of gene nodes and co-expression edges of the networks recorded for comparative analysis. The networks constructed with the unknown gene transcripts randomly selected from the ginseng transcriptome were used as the negative controls. The analysis was constructed 20 bootstrap replications. In addition, based on the connectivity of the *PgHDZ* genes in the co-expression networks, the *PgHDZ* genes that co-expressed with the largest number of genes and were closely correlated with the genes in the network were identified as the hub genes that play central roles in the networks.

### Roles of the *PgHDZ* genes in ginseng response to pH stress

One gram of ginseng adventitious roots were cultured in 250 mL liquid Gamborg B5 medium with an optimal pH = 6.0 for ginseng adventitious root growth at 22 °C, 110 RPM in dark for 30 days. On the 25^th^ day of cultivation, the pH value of the culture medium was adjusted to 4.0 and 5.0, respectively, using 0.01 M HCl for pH value decrease and to 7.0 and 8.0, respectively, using 0.01 M NaOH for pH value increase. Three biological replicates were performed for both the control group and each treatment group. The adventitious roots were continuously cultured for additional 5 days, then harvested and measured in fresh weight. One gram of the fresh roots of each sample were frozen in liquid nitrogen and stored at -80 °C for RNA isolation. The relative gene expressions of the *PgHDZ* genes were assayed by real-time quantitative PCR (RT-qPCR) according to Li et al. [55]. The RT-qPCR primers used to quantify relative expression of the five *PgHDZ* hub genes in the adventitious roots grown in the optimal pH (pH 6.0) condition and in the changed pH conditions are shown in Table S2. The Spearman’s correlation coefficients among the expressions of five hub genes were calculated by the IBM SPSS statistics software (version 23).

## Results

### Identification of the *PgHDZ* gene family and classification of its subfamilies

One hundred seventeen *HD-Zip* gene transcripts were identified from the ginseng transcriptome. Screening conserved domain protein sequences of the known *HD-Zip* genes against the ginseng transcriptome obtained 114 *HD-Zip* gene transcripts. Screening all *HD-Zip* genes at NCBI against the ginseng transcriptome identified 83 *HD-Zip* gene transcripts. The HMMER search resulted in 112 *HD-Zip* gene transcripts. Combining these *HD-Zip* gene transcripts and deleting those duplications yielded a total of 117* HD-Zip* gene transcripts. These transcripts had an average length of 1,885 bp and were designated as *PgHDZ* genes transcripts (Table S3). In comparison, the first and third search methods were more desirable than the second search method for *HD-Zip* identification, but neither of them could identify the 117 finalized *PgHDZ* gene transcripts. Only one of the 117 *PgHDZ* gene transcripts was specifically identified by the second search method. These results indicated that it was necessary to use multiple search methods to maximize the identification of the *PgHDZ* gene transcripts from the ginseng transcriptome.

The *PgHDZ* genes were classified into four subfamilies based on the conserved domain and motif contained in the transcripts (Table S3). Subfamily I included 73 transcripts; subfamily II consisted of 13 transcripts; subfamily III was made of 11 transcripts; and subfamily IV had 20 transcripts.

### The size of the *PgHDZ* gene family and its positions in the ginseng genome

As the *PgHDZ* gene transcripts were identified from the cv. Damaya transcriptome, it is difficult to estimate the size of the *PgHDZ* gene family. Therefore, the size of the gene family was estimated by means of the cv. ChP [1] and acc. IR826 [15] genomes. A total of 83 and 82 *HD-Zip* genes were identified from the cv. ChP and acc. IR826 genomes, respectively. Therefore, the *PgHDZ* gene family likely consists of approximately 80 genes, even though this size may vary across different genotypes.

**Fig. 1:**
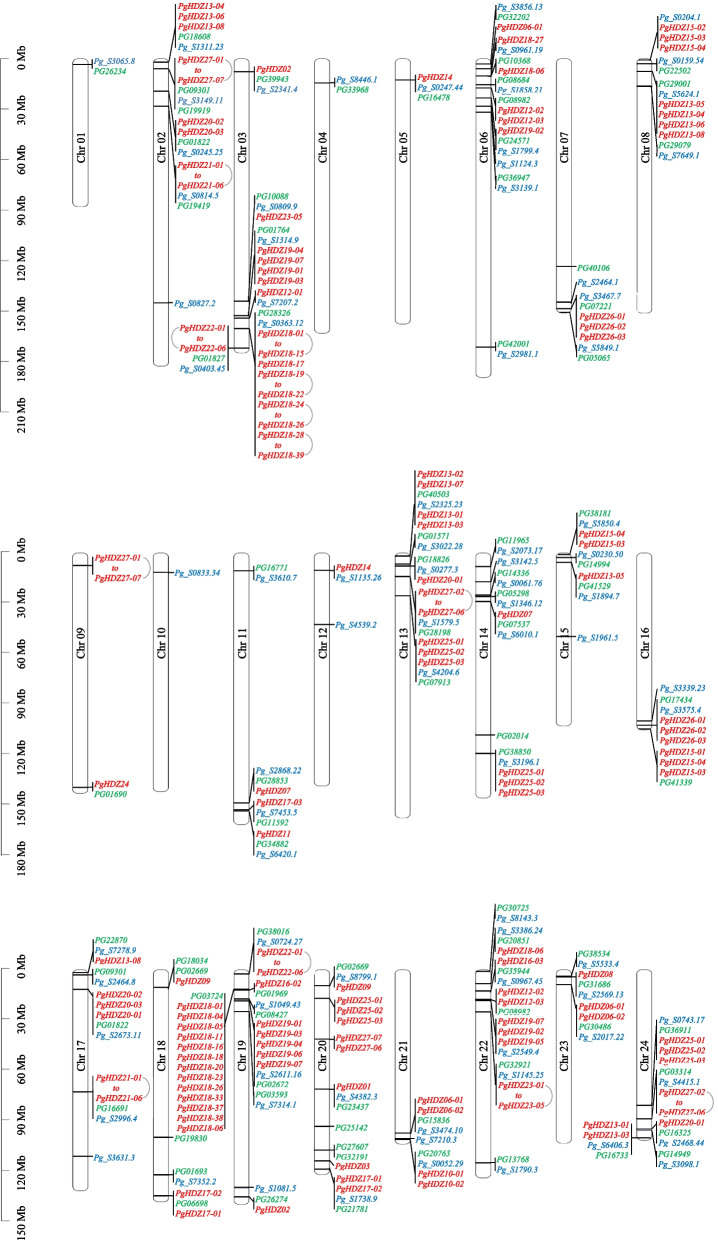
Distribution and positional variation of the *PgHDZ* gene family in the ginseng genome. The *PgHDZ* genes were mapped to all 24 ginseng chromosomes. The *PgHDZ* genes in blue font are from ginseng cv. ChP, the genes in green font are from acc. IR826, and the genes in red font are from cv. Damaya

The 117 *PgHDZ* gene transcripts from the cv. Damaya transcriptome, the 83 *HD-Zip* genes from the cv. ChP genome, and the 82 *HD-Zip* genes from the acc. IR826 genome were aligned to the 24 chromosomes of the ginseng genome (Fig.1). One hundred fourteen of the 117 *PgHDZ* gene transcripts from the cv. Damaya transcriptome were positioned to 64 loci of the ginseng genome, indicating that they were derived from 64 *PgHDZ* genes, while three could not be positioned to the ginseng genome, suggesting the diversity of the *PgHDZ* genes between genotypes. The 83 *HD-Zip* genes from the cv. ChP genome and the 82 *HD-Zip* genes from the acc. IR826 genome were aligned to the 83 and 82 loci of the ginseng genome, respectively, verifying the numbers of the *HD-Zip* genes in the genome. The *PgHDZ* or *HD-Zip* genes were distributed on all 24 chromosomes of the ginseng genome. Although it was difficult to determine which of the *HD-Zip* genes from the cv. ChP and acc. IR826 genomes are genotype-specific, due to the incompleteness of the cv. Damaya *PgHDZ* gene family, five cv. Damaya-specific *PgHDZ* genes were identified because they were positioned to unique loci of the ginseng genome.

### Conserved motifs and sequence structures of the *PgHDZ* gene transcripts

**Fig. 2:**
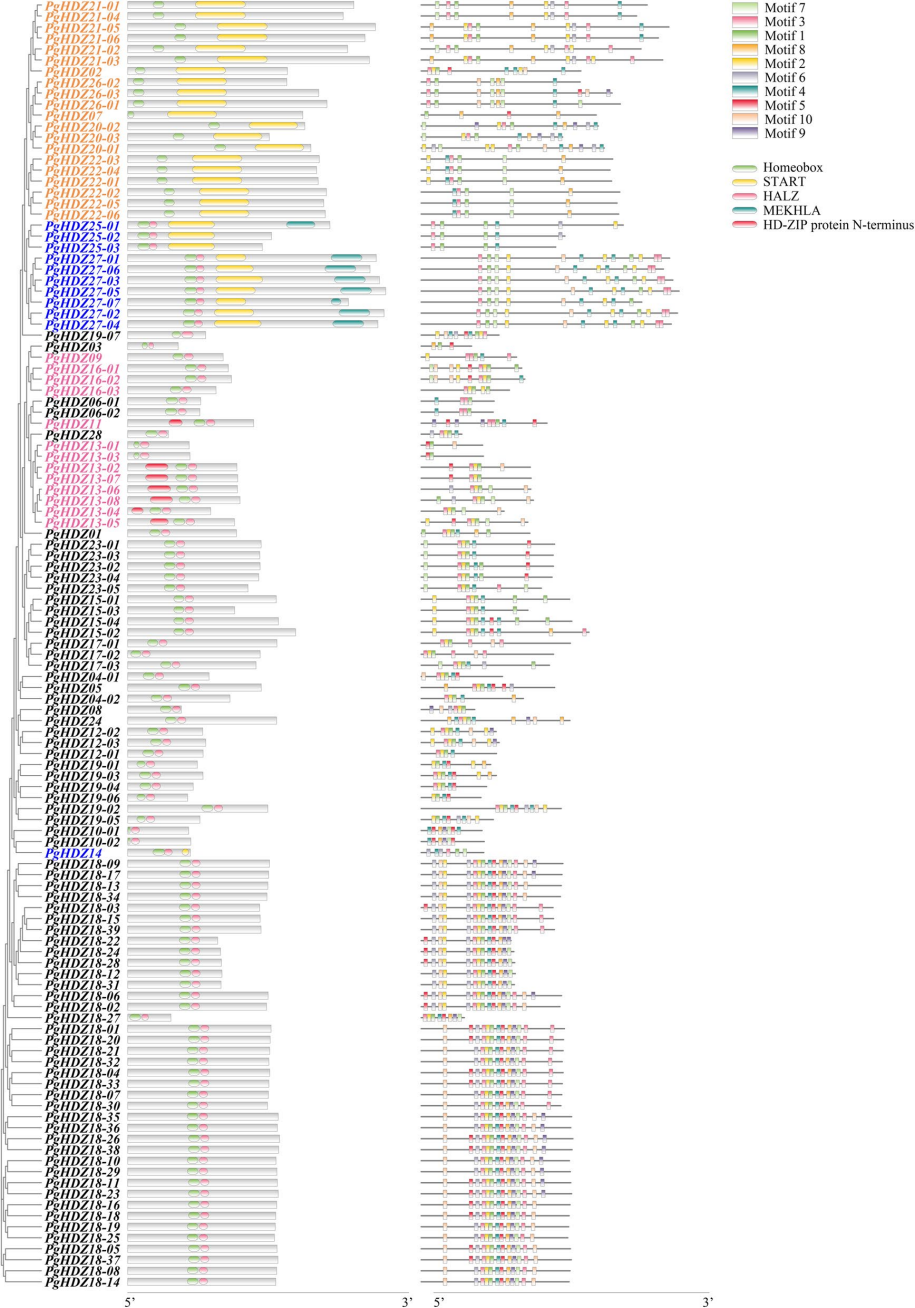
The structure of *PgHDZ* gene transcripts. The gene transcripts were clustered with MEGA in which the four subfamilies of the gene family are shown, with the transcripts in black font from subfamily I, the transcripts in pink font from subfamily II, the transcripts in blue font from subfamily III, and the transcripts in yellow font from subfamily IV. The conserved domains of the transcripts identified by NCBI Conserve Domain Search, including Homeobox, HALZ, START, MEKHLA, and the HD-Zip protein N-terminus, are shown in the left column and the conserved motifs of the transcripts identified by MEME, including motifs 1 through 10, are shown in the right column. For details of the 10 conserved motifs, see Fig. S1

Ten conserved motifs with a length of 50 nucleotides, designated motif 1 through motif 10, were identified from the *PgHDZ* gene transcripts (Fig. S1). Figure 2 shows the distribution of these motifs in the *PgHDZ* gene transcripts. The average number of motifs contained in each *PgHDZ* gene transcripts was 9.9 motifs per transcript for those subfamily I, 5.9 motifs per transcript for those subfamily II, 9.4 motifs per transcript for those subfamily III, and 7.3 motifs per transcript for those subfamily IV. Among the 10 conserved motifs, motifs 4, 5, and 6 were extraordinarily conserved, and motifs 1, 2, and 3 were relatively less conserved, but contained in more *PgHDZ* gene transcripts.

Five conserved domains, including homeobox, START, HALZ, MEKHLA and HD-Zip protein N-terminus, were identified from the *PgHDZ* gene transcripts (Fig. 2). The genes of subfamily I all contain only the homeobox and HALZ domains. Some of the genes in subfamily II contain the homeobox and HALZ domains, and HD-Zip protein N-terminus, but *PgHDZ09* and *PgHDZ16* of the subfamily contain only the homeobox and HALZ domains. The genes of subfamily III have the homeobox, START, HALZ domains, but *PgHDZ25* and *PgHDZ27* contain another domain, MEKHLA. All subfamily IV genes contain the homeobox and START domains.

### Phylogeny and synonymous and non-synonymous substitution rates of the *PgHDZ* gene family

**Fig. 3:**
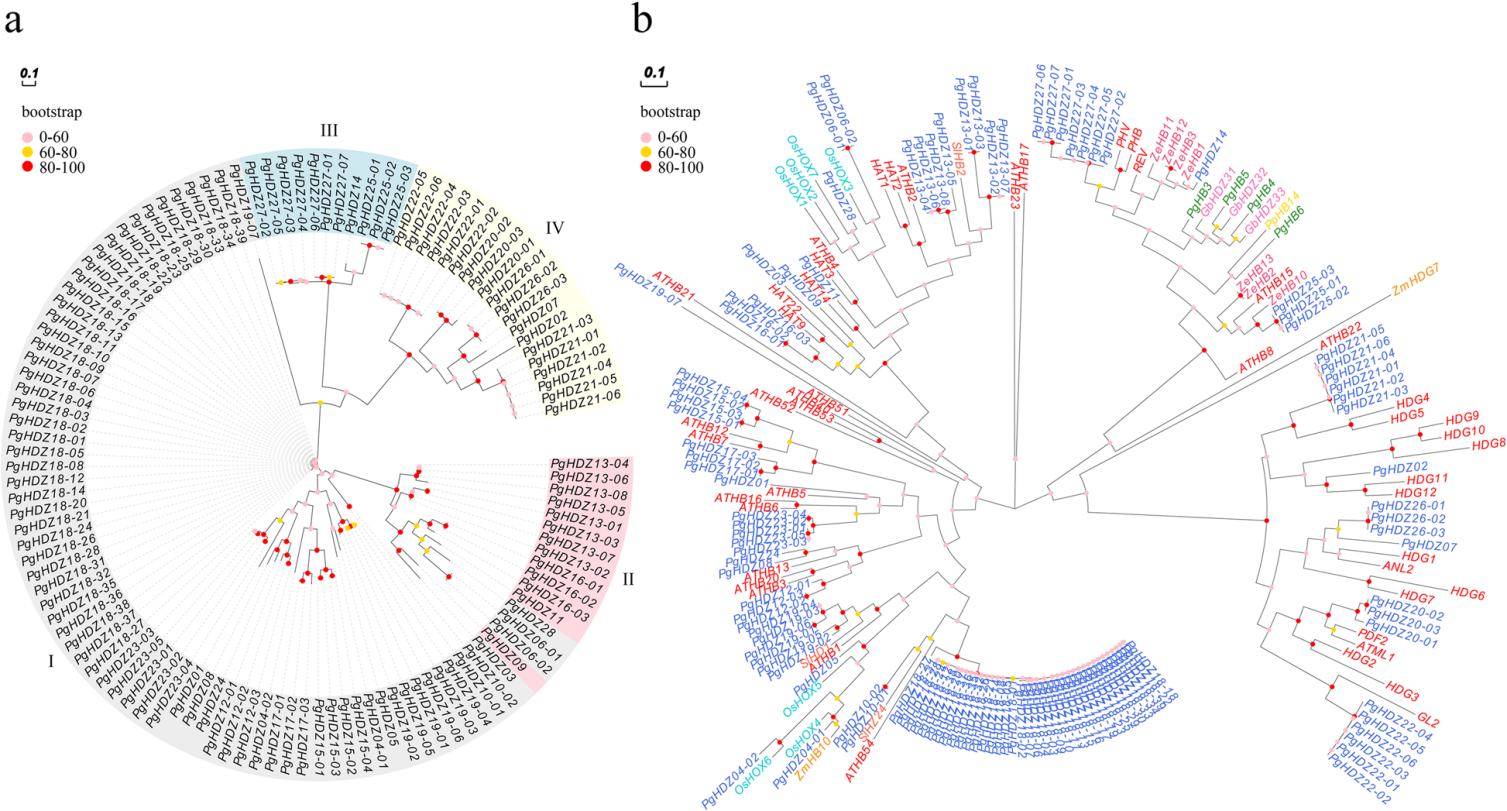
The phylogenetic trees of the *PgHDZ* gene family. **a** The phylogenetic tree of the *PgHDZ* gene transcripts constructed without the *HD-Zip* genes from outgroup species. The subfamilies of the gene family are indicated by I, II, III, and IV, and highlighted with different colored backgrounds. **b** The phylogenetic tree of the *PgHDZ* gene transcripts constructed with the *HD-Zip* genes from outgroup species. The *HD-Zip* genes from different species are highlighted in different colored fonts. The genes in blue font are from *Panax ginseng* (*Pg*); the gene in dark yellow from *P. patens* (*Pp*) (Moss); the genes in green font from *Picea glauca* (*Pg*) (Gymnosperm); the genes in pink font from *G. biloba* (*Gb*) (Gymnosperm); the genes in light-blue font from *O. sativa* (*Os*) (Monocots); the genes in orange font from *Z. mays* (*Zm*) (Monocots); the genes in red font from *A. thaliana* (*At*) (Eudicots-Rosids); the genes in dark pink from *Z. elegans* (*Ze*) (Eudicots-Asterids); and the genes in dark orange from *S. lycopersicum* (*Sl*) (Eudicots-Asterids). The trees were conducted with the Maximum Likelihood method by MEGA, with 2000 bootstrap replications. The circle in each branch indicates its confidence in percentage. The bar in the left-top corner of each tree indicates the scale of the tree branch lengths

The phylogenetic tree of the *PgHDZ* family was first constructed, without using the *HD-Zip* genes identified from other species as outgroup (Fig. 3a). The tree consisted of four clear clusters that corresponded to the four subfamilies of the *PgHDZ* gene family. The clustering completely agreed with the subfamily classification of the family [56], except for one of the gene, *PgHDZ09*, in the subfamily II was clustered with the genes of subfamily I. Moreover, the tree also revealed that subfamilies III and IV more diverged than subfamilies I and II.

Then, the phylogenetic tree of the *PgHDZ* gene family was constructed using *HD-Zip* genes identified from an evolutionary range of taxa as out-groups to infer the origin and evolution of the gene family (Fig. 3b). These taxa included Moss (*Physcomitrella patens*), Gymnosperm (P*icea glauca* and *Ginkgo biloba*), Monocot (*Oryza sativa* and *Zea mays*), Eudicots-Rosides (*Arabidopsis thaliana*), and Endicots-Asterids (*Zinnia elegans* and *Solanum lycopersicum*) [57] (Table S1). The phylogenetic tree shows that the *PgHDZ* genes were clustered not only with the *HD-Zip* genes contained in Dicots (*A. thaliana*, *Zinnia elegans*, and *S. lycopersicum*), but also with those contained in Monocots (*O. sativa* and *Zea mays*). Nevertheless, none of the *PgHDZ* genes was clustered with the *HD-Zip* genes contained in Gymnosperm (*P. glauca* and *G. biloba*) and Moss (*P. patens*). These results indicated that the *PgHDZ* gene family originated and evolved after Angiosperm split from Gymnosperm, but before Monocots split from Dicots.

To estimate when the latest duplication of the *PgHDZ* gene family occurred, two gene pairs of the *PgHDZ* gene family, *PgHDZ15*/*PgHDZ17* and *PgHDZ09*/*PgHDZ16*, that likely duplicated most recently were used to calculate the Ka/Ks ratio (Table 1). Both *PgHDZ15* and *PgHDZ17* belonged to subfamily I and both *PgHDZ09* and *PgHDZ16* belonged to subfamily II. The Ka/Ks ratios of *PgHDZ15*/*PgHDZ17* and *PgHDZ09*/*PgHDZ16* were 0.563 and 0.415, respectively. These results suggested that these two pairs of genes diverged in 27 MYA (million years ago) and 29 MYA, respectively, and had been subjected to purifying selection.

**Table 1:** Gene duplications, synonymous (Ka) and non-synonymous (Ks) substitutions and time of divergence of the *PgHDZ* gene family

Duplicated gene pairs		Ka	Ks	Ka/Ks	Date (Million Years Ago)
*PgHDZ15-02*	*PgHDZ17-01*	0.205	0.364	0.563	27.981
*PgHDZ09*	*PgHDZ16-03*	0.159	0.383	0.415	29.481

### Annotation and ontology of the *PgHDZ* gene family

**Fig. 4:**
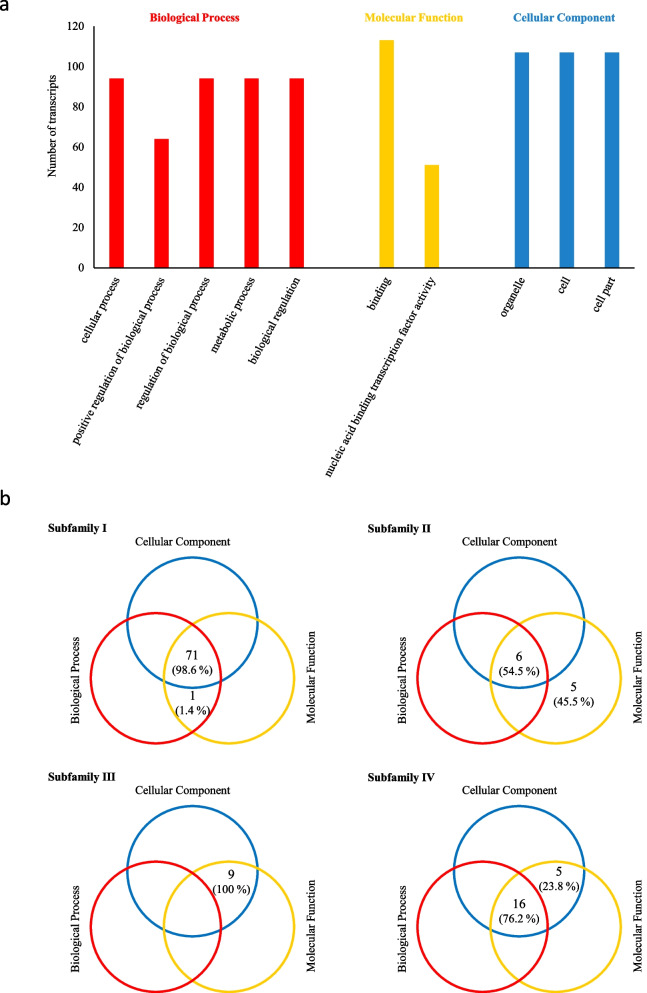
Annotation and GO categorization of the *PgHDZ* gene family. **a** The GO categories of the *PgHDZ* gene transcripts (Level 2). **b** The number of *PgHDZ* gene transcripts categorized into Biological Process, Molecular Function, and Cellular Component from different subfamilies of the *PgHDZ* gene family

To have a general knowledge about the genes of the *PgHDZ* gene family, the 117 transcripts of the *PgHDZ* genes were annotated and categorized with gene ontology (GO). One hundred thirteen of the 117 transcripts were annotated and categorized into all three primary categories, Biological Process (BP), Molecular Function (MF), and Cellular Component (CC) (Fig. 4a). In the MF category, 113 *PgHDZ* genes transcripts were involved in binding and 51 transcripts were involved in nucleic acid binding transcription factor activity. In the BP category, 94 transcripts participated in cellular process, regulation of the biological process, metabolic process, and biological regulation, and 64 transcripts played roles in positive regulation of the biological process. In the CC category, 109 transcripts functioned for organelle, cell, and cell part. These GO results of the *PgHDZ* gene family are in accord with the functions of the transcription factors.

Of the 113 *PgHDZ* gene transcripts, 72 were from subfamily I, of which 71 were categorized into all three primary categories, BP, MF, and CC, and one categorized into BP and MF (Fig. 4b). Eleven of the 113 transcripts were from subfamily II, of which six were categorized into BP, MF, and CC, and five were MF-specific. Nine of the 113 transcripts were from subfamily III and all nine transcripts were categorized into MF and CC. Twenty-one of the 113 transcripts were from subfamily IV, of which 16 were categorized into BP, MF, and CC, and five categorized into MF and CC. These results indicated that the functions of *PgHDZ* genes have diverged substantially between subfamilies.

### Spatiotemporal expressions of *PgHDZ* gene transcripts and their expression diversity among cultivars

**Fig. 5:**
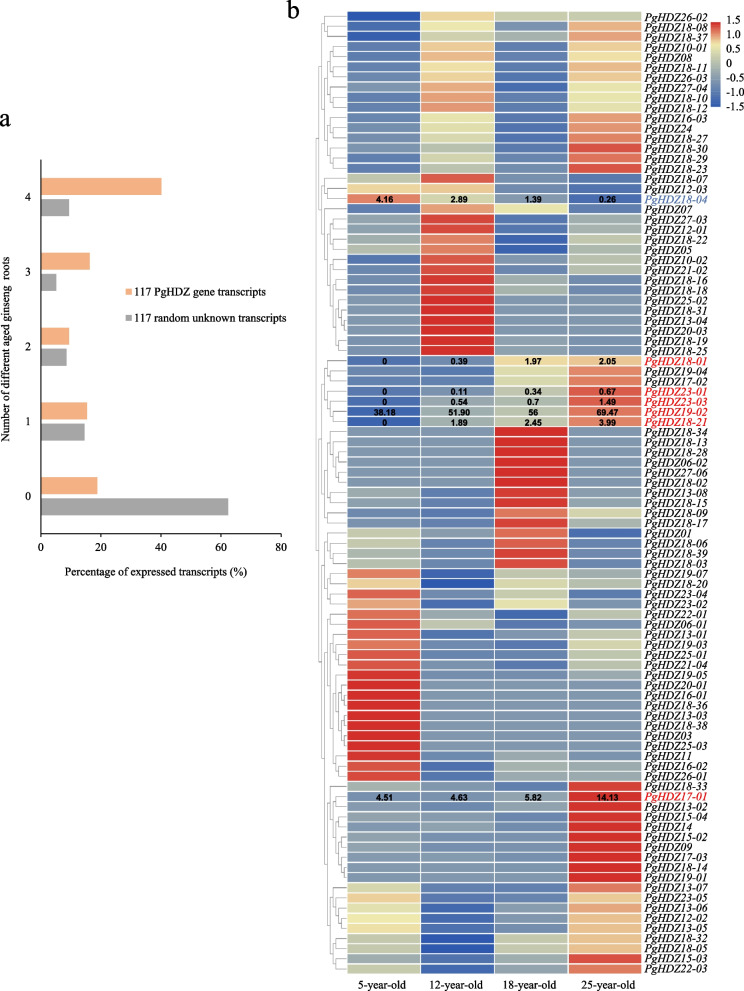
Expressions of the 117 *PgHDZ* gene transcripts in different year-old ginseng roots. **a** Percentage of *PgHDZ* transcripts expressed in four different aged ginseng roots with 117 transcripts randomly selected from the ginseng transcriptome as the reference. **b** Expression heatmap of *PgHDZ* transcripts in different year-old ginseng roots. The transcripts whose expressions varied regularly with root ages are highlighted in red or blue font, and their expression levels (TPM) are indicated in the heatmap

The expressions of the *PgHDZ* gene transcripts were assayed at different developmental stages of plant roots, in different tissues of a four-year-old plant, and in four-year-old plant roots of different cultivars to characterize their expression patterns and diversity among cultivars. Of the 117 *PgHDZ* gene transcripts, 95 (81.2%) expressed in at least one of the 5-, 12-, 18-, and 25-year-old plant roots. Eighteen (15.4%), 1 (9.4%), 19 (16.2%), and 47 (40.0%) of the 117 gene transcripts expressed in one, two, three, and all of the four different aged plant roots, respectively. In comparison, only approximately 40% of the 117 unknown gene transcripts randomly selected from the ginseng transcriptome expressed in at least one of the different aged plant roots (Fig. 5a), which was over 100% fewer than the number of the *PgHDZ* gene transcripts in the different aged plant roots. The expression heatmap of the transcripts showed that most of the *PgHDZ* transcripts actively expressed at one of the four developmental stages, but some of the transcripts indeed actively expressed at two of the four developmental stages such as in the 12-year-old and 25-year-old or 18-year-old and 25-year-old plant roots (Fig. 5b). Interestingly, the expression activities of some of the transcripts exhibited regular variation with the plant growth and development, including the decrease of expression activity as the plant age increased, such as *PgHDZ18-04* and the increase of expression activity as the plant age increased, such as *PgHDZ17-01*, *PgHDZ18-01*, *PgHDZ18-21*, *PgHDZ19-02*, *PgHDZ23-01*, and *PgHDZ23-03*. These seven genes might participate in some particular biological processes at ginseng different growth and development stages and could be used as candidate gene markers for ginseng age identification and/or validation.

**Fig. 6:**
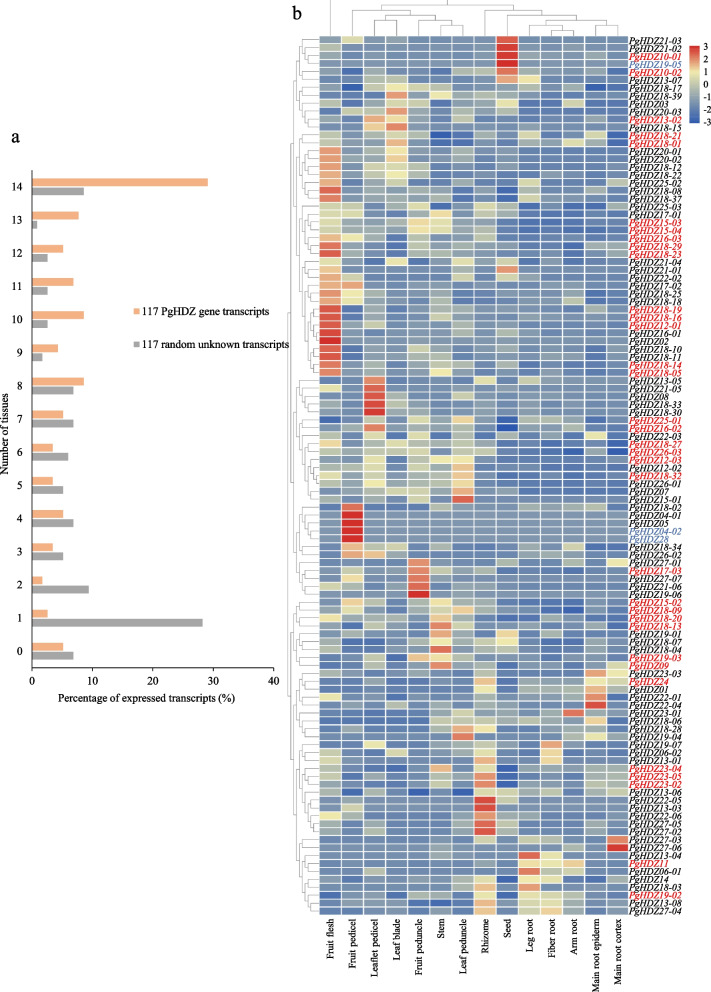
Expressions of the 117 *PgHDZ* gene transcripts in different tissues of a four-year-old plant. **a** Percentage of *PgHDZ* transcripts expressed in 14 tissues with 117 transcripts randomly selected from the ginseng transcriptome as the reference. **b** Expression heatmap of *PgHDZ* transcripts in different tissues. The transcripts that expressed in all 14 tissues and in only one tissue are highlighted in red and blue fonts, respectively

At the spatial expression, 111 (94.9%) of the 117 *PgHDZ* transcripts expressed in at least one of the 14 tissues of a four-year-old ginseng plant. Particularly, 34 (29.1%) of the 117 transcripts expressed in all 14 tissues, which was much higher than the 8.6% of the unknown transcripts randomly selected from the ginseng transcriptome (Fig. 6a). Further analysis showed that only three of the transcripts expressed in one of the 14 tissues, including *PgHDZ04-02* and *PgHDZ28* expressed in fruit pedicel, and *PgHDZ19-05* expressed in seed. These three transcripts might play important roles in ginseng fruit pedicel and seed development. The expression heatmap of these 111 *PgHDZ* transcripts clustered the 14 tissues of the four-year-old plant into two groups, above-ground (stem, leaf peduncle, leaflet pedicel, leaf blade, fruit peduncle, fruit pedicel, and fruit flesh) and under-ground (fiber root, leg root, main root epiderm, main root cortex, rhizome, and arm root), with an exception for seed that was above-ground, but clustered into the under-ground group (Fig. 6b). Approximately 70% of the 117 transcripts expressed higher in the above-ground tissues than in the under-ground tissues, especially in the fruit flesh. These results indicated that the *PgHDZ* gene family may play more important roles in the formation and development of stem, leaf, and fruit than in those of root systems and seed.

Expression analysis of the *PgHDZ* gene transcripts across cultivars revealed that 108 (92.3%) of the 117 transcripts expressed in the four-year-old plant roots of at least one of the cultivars, but only 24 (20.5%) of them expressed in the four-year-old plant roots of all 42 cultivars (Fig. S2a). The expression heatmap of these 108 transcripts showed that only a limited number of the transcripts actively expressed in the four-year-old plant root of each cultivar (Fig. S2b). This result may spotlight that the *PgHDZ* gene transcripts are mainly responsible for response to environmental changes. Furthermore, 19 of the *PgHDZ* transcripts were found to express in all aged plant roots, all 14 tissues, and the four-year-old plant roots of all 42 cultivars (Fig. S3). These 19 *PgHDZ* gene transcripts may be essential for ginseng growth and development.

### Co-expression networks of the *PgHDZ* gene transcripts and their potential hub-genes

To estimate the relationships of the genes in the *PgHDZ* gene family in functionality, their co-expression networks were constructed using their expressions in the four-year-old roots of 42 cultivars. All 108 *PgHDZ* transcripts expressed in one or more of the 42 cultivars formed a co-expression network with 10 clusters and 826 co-expression edges at a *p*-value of 5.0E-02 (Fig. S4a and b). Comparative analysis showed that the network constructed with the *PgHDZ* gene transcripts had substantially more nodes and co-expression edges than that constructed with 108 unknown transcripts randomly selected from the ginseng transcriptome at any *p*-value varying from 5.0E-02 to 1.0E-08 (Fig. S4c and d). Moreover, the differences of these networks in number of nodes and number of edges were tested statistically using the bootstrap samples of the transcripts as replications. Both the number of nodes and number of edges of the *PgHDZ* gene transcripts were significantly higher than those of the randomly selected unknown transcripts at any *p*-value varying from 5.0E-02 to 1.0E-08 (Fig. S4e and f), thus confirming that the *PgHDZ* gene transcripts were much more likely to form a co-expression network than those randomly selected transcript controls. These results indicated that the gene members of the *PgHDZ* gene family remained correlated in functionality.

**Fig. 7:**
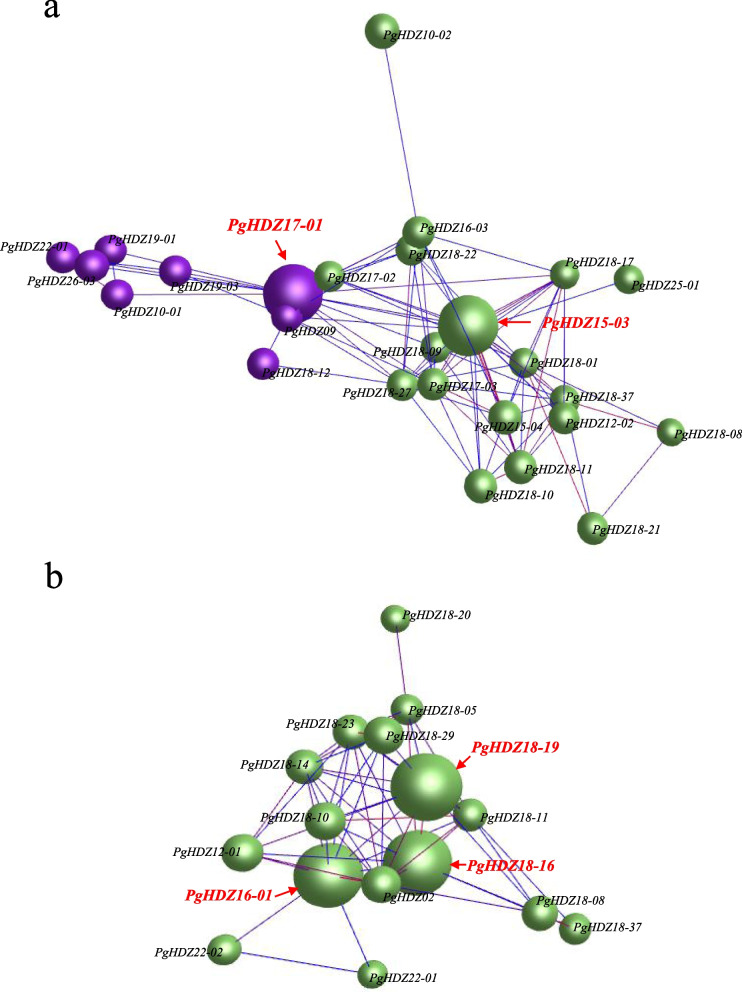
The potential hub genes identified from the co-expression network of the *PgHDZ* gene family. **a** Cluster 01 and cluster 02, indicated by different colors, of the *PgHDZ* gene family co-expression network in four-year-old plant roots of 42 cultivars. The co-expression network was constructed at *p* ≤ 1.0E-03 and the potential hub genes that were correlated with the largest number of the genes and with a connectivity of larger than 15 are highlighted in red font. **b** Cluster 01 of the *PgHDZ* gene family co-expression network in different tissues of a four-year-old ginseng plant. The co-expression network was constructed at *p* ≤ 1.0E-03 and the potential hub genes that were correlated with the largest number of genes and with a connectivity of larger than 10 are highlighted in red font

Finally, the network was re-constructed at a *p*-value ≤ 1.0E-03 to identify the hub gene(s) of the network that play central roles, particularly having the largest number of genes with which it interacted in the network. Based on this criterion, *PgHDZ15-03* and *PgHDZ17-01* were identified as the potential hub genes in the four-year-old plant roots of 42 cultivars (Fig. 7a). Similarly, a network was also constructed at a *p*-value ≤ 1.0E-03 to identify the hub gene(s) of the network of the *PgHDZ* gene transcripts in 14 tissues of the four-year-old plant. It was apparent that *PgHDZ16-01*, *PgHDZ18-16*, and *PgHDZ18-19* were the potential hub genes in the network (Fig. 7b).

### The role of the *PgHDZ* genes in plant response to pH stress

**Fig. 8:**
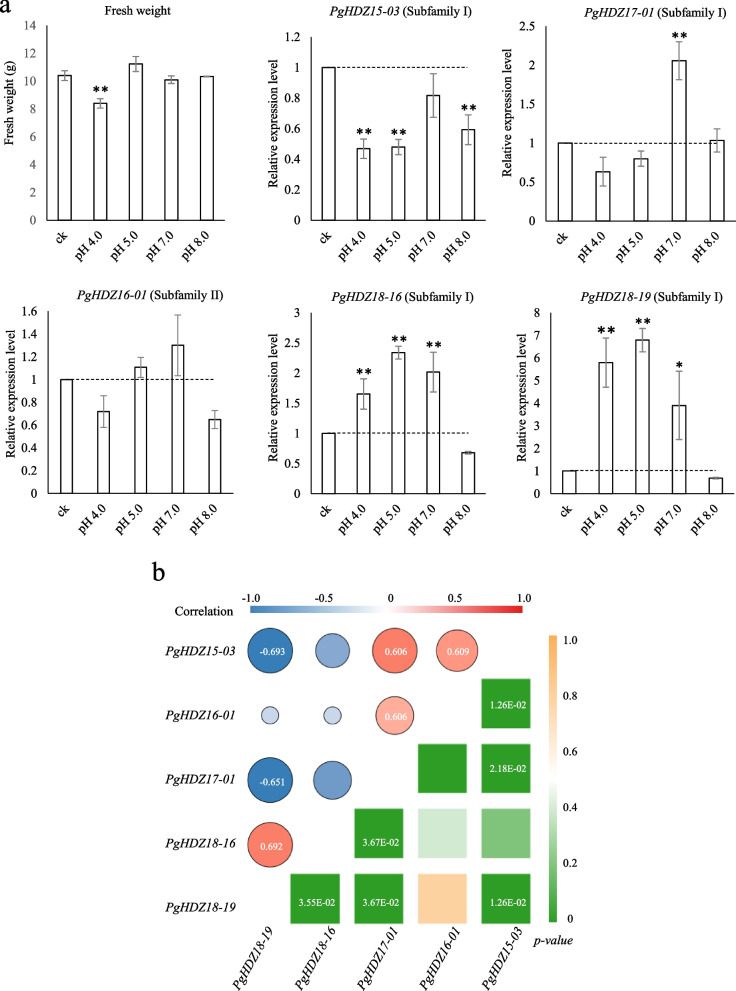
Impacts of pH stress on ginseng adventitious root growth and the responses of the *PgHDZ* hub genes to the stress. **a** Impacts of pH stress on adventitious root fresh weight and the relative expressions of the five *PgHDZ* hub genes to the stress. “*” for a two-tailed significance of *p* ≤ 5.0E-02 and “**” for a two-tailed significance of *p* ≤ 1.0E-02. **b** The Spearman’s correlation coefficients among the expressions of the five *PgHDZ* hub genes in responses to pH stress

Ginseng is adapted for growth and development in a soil with pH = 6.0. Therefore, the pH value was adjusted to 5.0 and 4.0 by adding HCl or to 7.0 and 8.0 by adding NaOH, respectively, to the culture medium. On 5^th^ day after the pH value was adjusted, the fresh weight of cultured adventitious roots significantly decreased for the roots on the medium with pH 4.0, while that of the cultured roots with pH 5.0, 7.0, and 8.0 had no significant change (Fig. 8a). This result indicated that ginseng adventitious roots were sensitive to low pH stress, but were not or less sensitive to higher pH stresses. Moreover, the expressions of the five hub genes identified above, including *PgHDZ15-03*, *PgHDZ17-01*, *PgHDZ16-01*, *PgHDZ18-16*, and *PgHDZ18-19*, were examined by RT-qPCR. The hub genes, *PgHDZ15-03* and *PgHDZ17-01*, in the network of the *PgHDZ* genes in the four-year-old roots of different cultivars had opposite responses to the medium pH change, with the expression of *PgHDZ15-03* decreasing when the medium pH changed to pH 4.0, 5.0 and 8.0, while the expression of *PgHDZ17-01* increasing when the medium pH was changed to pH 7.0 (*p* ≤ 1.0E-02). The hub gene, *PgHDZ16-01*, in the network of different tissues of the four-year-old plant exhibited no significant change in expression, and the expressions of *PgHDZ18-16* and *PgHDZ18-19* were increased when the medium pH was changed to pH 4.0, 5.0, and 7.0 (*p* ≤ 1.0E-02) (Fig. 8a). Furthermore, the expression of *PgHDZ15-03* was positively correlated with those of *PgHDZ16-01* and *PgHDZ17-01* and negatively correlated with that of *PgHDZ18-19* (*p* ≤ 5.0E-02). The expression of *PgHDZ18-19* was positively correlated with those of *PgHDZ18-16* and negatively correlated with *PgHDZ17-01* (*p* ≤ 5.0E-02). The expressions of *PgHDZ16-01* and *PgHDZ17-01* were positively correlated (*p* ≤ 5.0E-02) (Fig. 8b). These results indicated that the *PgHDZ* genes, including *PgHDZ15-03*, *PgHDZ17-01*, *PgHDZ18-16,* and *PgHDZ18-19*, play important roles in regulation of plant response to environmental pH change and their interaction may reflect at least in part their molecular mechanism in regulating plant response to environmental pH variation.

## Discussion

The *HD-Zip* gene family has been documented to play vital roles in plant growth and development and plant responses to abiotic stresses, but it has not been studied in ginseng and related species. The present study has identified 117 transcripts of the *HD-Zip* genes, designated as *PgHDZ* gene transcripts, from the transcriptome of the Jilin ginseng cv. Damaya that is widely grown in Jilin, China – the origin, diversity, and production center of ginseng. These *PgHDZ* gene transcripts are positioned to 64 loci of 21 of the 24 ginseng chromosomes. Since the use of transcriptome for the gene family identification limited the proper estimation of the family size, identification of the *HD-Zip* genes for ginseng was further carried out using the draft genomes of ginseng cv. ChP and acc. IR826, from which 83 and 82 *HD-Zip* genes were identified, respectively. These *HD-Zip* genes were positioned to 83 loci of 23 of the 24 ginseng chromosomes and 82 loci of 22 of the 24 ginseng chromosomes. This result indicates that the *PgHDZ* gene family consists of approximately 80 gene members. This gene family size is much larger than those of the *HD-Zip* gene family in pepper (40) [58], maize (55) [59], Arabidopsis (48) [27], and grape (33) [60]. The larger size of the *PgHDZ* gene family is consistent with its specific expansion found in the ginseng genome [1], probably suggesting its essential functions in the ginseng species.

Phylogenetic analysis has shown that the *PgHDZ* gene family is an ancient gene family that originated after Angiosperm split from Gymnosperm and before Dicots split from Monocots. Since then, the gene family has expanded substantially, with the latest gene duplication occurred between 27 and 29 MYA. The gene members of the family have been diverged dramatically at the nucleotide sequence level, at positions in the genome, and in corresponding functionality, even though the feature domains of the gene family largely remain conserved. For instance, the genes of the family were annotated and categorized into all three GO primary categories and 10 GO subcategories (Level 2), varying from cellular processes to binding and cell part, based on sequence similarities. Of the 64 chromosomal loci of the 117 *PgHDZ* gene transcripts, five are Damaya-specific and three gene transcripts were not found in the ginseng reference genome. Both phylogenetic analysis and present/absence of conserved domains classified the *PgHDZ* gene family into four subfamilies, leading to that different subfamilies have different conserved domains and motifs.

Furthermore, the functional differentiation of the *PgHDZ* genes has been also revealed at the gene expression level in different tissues of a plant, across developmental stages, and across cultivars. For instance, only 19 (16%) of the 117 *PgHDZ* gene transcripts were found to express in all four aged plant roots, all 14 tissues of a four-year-old plant, and in the four-year-old plant roots of all 42 cultivars. These transcripts may play indispensable roles in ginseng growth and development. Fourteen of the 19 gene transcripts are from subfamily I, four from subfamily II, one from subfamily III, and none from subfamily IV. Because the *HD-Zip* gene family, especially subfamily I, was reported to have the function of plant response to abiotic stresses in plants [37], the consistent expressions of the transcripts may imply the important function of subfamily I in plant basic biological processes. In addition, seven of the *PgHDZ* gene transcripts were found to have special expression patterns that changed as the ginseng plant age increased and three were found to express only in a particular tissue. These transcripts have the potential to serve as candidate gene markers for the quality identity of ginseng products. Nevertheless, the present study shows that the genes of the *PgHDZ* gene family form a single co-expression network, suggesting that the genes of the family remain correlated in functionality. This result is in consistence with the previous studies showing that the *HD-Zip* genes tended to interact with each other [39] or with other transcription factor genes [61]. It has been shown that the complex formed with *HD-Zip* genes and other transcription factor proteins had roles in regulating plant tissue development, such as leaf rolling of rice [62], glandular secretory trichome initiation in *Artemisia annua* [61], and the size of spine base in cucumber [63]. Therefore, the *PgHDZ* genes may also form a complex with other transcription factor proteins to regulate plant growth and development in ginseng.

Continuous cropping has been found to lead to the decline of genus *Panax* yield and quality. It has been reported that the soil pH was lower in the field planted with ginseng than in the field not planted with ginseng; therefore, the declined ginseng yield and quality were associated with continuous monocropping [64]. The present study has verified this conclusion. When the culture medium pH was lowered to 4.0, the fresh weight of ginseng adventitious roots significantly decreased. The result indicates that ginseng is sensitive to low pH and improving ginseng resistance to low pH condition may alleviate the continuous cropping barrier for ginseng production. In this study, five hub genes were identified for the *PgHDZ* gene family, of which four were shown to significantly respond to culture medium pH changes. These genes are useful for deciphering the molecular mechanism of ginseng response to the soil pH change, thereby laying a foundation for improving the ginseng resistance to soil pH change and mitigating the problem of continuous cropping in ginseng production.

## Conclusions

The *PgHDZ* gene family was identified and shown to consist of approximately 80 genes. The *PgHDZ* gene family likely originated after Angiosperm split from Gymnosperm and before Dicots split from Monocots. The family was classified into four subfamilies and these subfamilies have diverged greatly in nucleotide sequence, chromosomal position, and GO annotation and categorization. Although the expressions of the gene family members varied dramatically in different year-old plant roots, different tissues of a four-year-old plant, and four-year-old plant roots across cultivars, genes of the family remain correlated in functionality. Five hub *PgHDZ* genes were identified to play central roles in ginseng biological processes and four of them were verified to regulate plant response to pH stress in ginseng.

## Supplementary Information

**Additional file 1: Fig. S1.** The conserved motifs of the genes in the *PgHDZ* gene family determined by MEME. The height of each letter represents the conservation level of the nucleotide across the *PgHDZ* genes.

**Additional file 2: Fig. S2.** Expressions of the 117 *PgHDZ* gene transcripts in four-year-old plant roots of 42 cultivars. (a) Percentage of *PgHDZ* transcripts expressed in 42 cultivars with 117 transcripts randomly selected from the ginseng transcriptome as the reference. (b) Expression heatmap of the *PgHDZ* transcripts in different cultivars.

**Additional file 3: Fig. S3.** The average expression of the 19 *PgHDZ* transcripts expressed in all 42 cultivars, 14 tissues and four aged roots. The genes belonging to different subfamilies were shown, with the genes black font from subfamily I, the genes in pink font from subfamily II, and the genes in blue font from subfamily III.

**Additional file 4: Fig. S4.** The co-expression network of the *PgHDZ* gene transcripts expressed in four-year-old plant roots of 42 cultivars. (a) The co-expression network of the *PgHDZ* gene transcripts constructed at *p* ≤ 5.0E-02. (b) Ten clusters of the co-expression network. (c and d) The co-expression network formation tendency of the *PgHDZ* gene transcripts presented by the number of nodes (c) and the number of edges (d). (e and f) Statistics of the co-expression network formation tendency of the *PgHDZ* gene transcripts presented by the number of nodes (e) and the number of edges (f). The 100 *PgHDZ* and unknown transcripts were randomly selected with 20 bootstraps. The capital letter represents that the difference between *PgHDZ* and random unknown transcripts was significant at *p* ≤ 1.0E-02.

**Additional file 5: Table S1.** The *HD-Zip* genes of the outgroup species used in the phylogenetic tree of the *PgHDZ* gene family.

**Additional file 6: Table S2.** Primers used for relative expression analysis of *PgHDZ* hub genes in the adventitious roots subjected and not subjected to pH stress by RT-qPCR.

**Additional file 7: Table S3.** The nucleotide sequences of the *PgHDZ* gene transcripts identified from ginseng cv. Damaya and their subfamilies.

## Data Availability

All the supporting data are included within the article and its additional files.

## Abbreviations

HD-Zip: homeodomain leucine zipper
cv: cultivar
acc: accession
ChP: Chunpoong
GO: gene ontology
pH: potential of hydrogen
TFs: transcription factors
bHLH: basic helix-loop-helix
SPL: SQUAMOSA promoter binding protein-like
MYB: v-myb avian myeloblastosis viral oncogene homolog
HD: homeodomain
HALZ: homeobox-associated leucine zipper
CTR: carboxy-terminal region
NTR: amino-terminal region
bZIP: basic leucine zipper
*P. ginseng*: *Panax ginseng*
NCBI: National Center for Biotechnology Information
START: STeroidogenic Acute Regulatory protein-related lipid Transfer
PFAM: protein families database
MEME: Multiple Expectation Maximization for Motif Elicitation
MEGA: Molecular Evolutionary Genetics Analysis
Ka: synonymous substitution rate
Ks: non-synonymous substitution rate
BP: Biological Process
MF: Molecular Function
CC: Cellular Component
RT-qPCR: real-time quantitative polymerase chain reaction
MYA: million years ago
TPM: Transcripts Per Million.
