# Supplementary material for: Genome-wide identification and systematic analysis of the HD-Zip gene family and its roles in response to pH in Panax ginseng Meyer
Source: BMC Plant Biol. 2023 Jan 13;23:30. doi: 10.1186/s12870-023-04038-9 (PMC9838044; doi:10.1186/s12870-023-04038-9)
Supplement: Supplementary file 1 — Additional file 1: Fig. S1. The conserved motifs of the genes in the PgHDZ gene family determined by MEME. The height of each letter represents the conservation level of the nucleotide across the PgHDZ genes. [file 12870_2023_4038_MOESM1_ESM.pptx]

## Slide 1
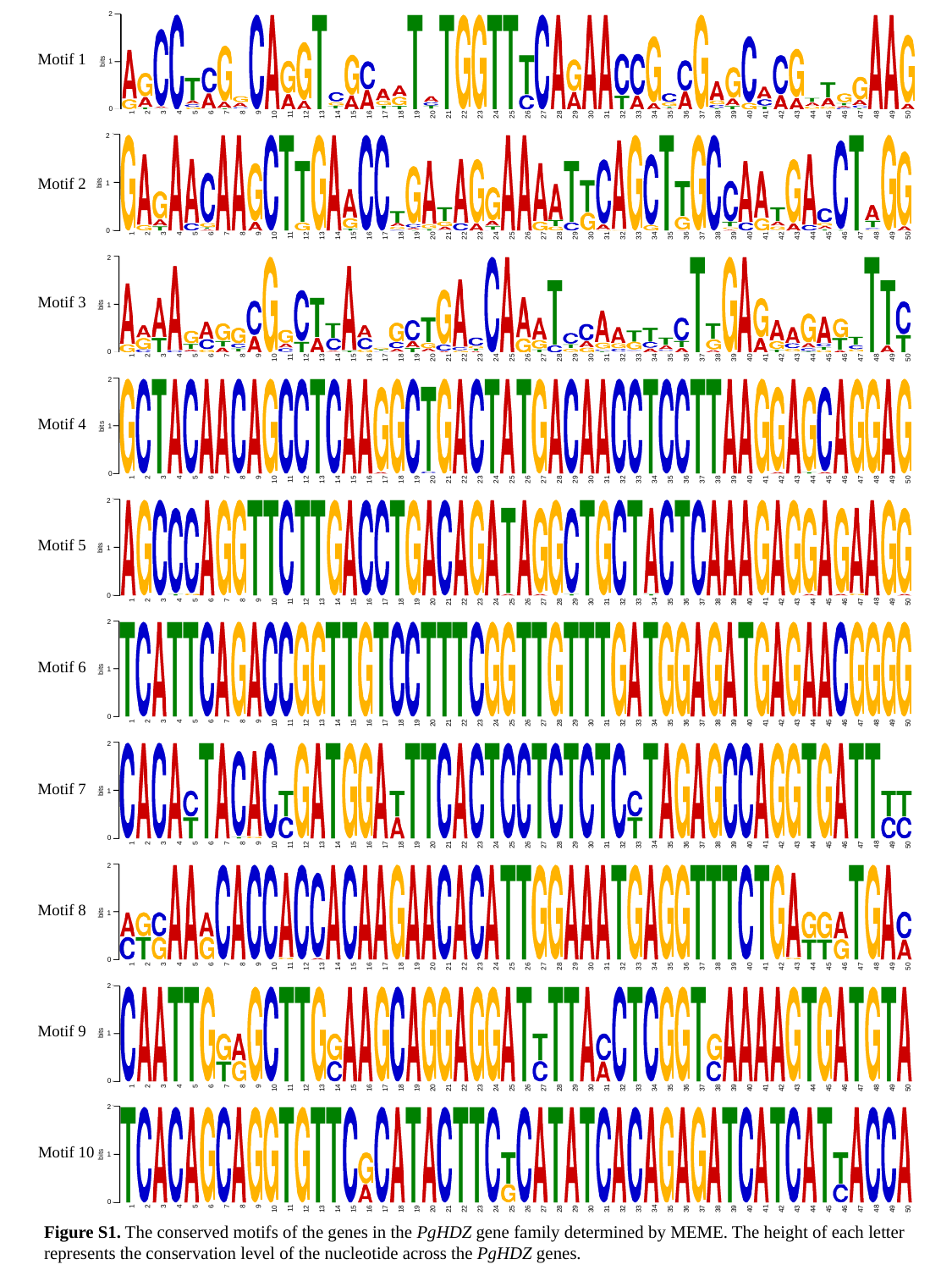

2
Motif 1
1
bits
0
1
2
3
4
5
6
7
8
9
10
11
12
13
14
15
16
17
18
19
20
21
22
23
24
25
26
27
28
29
30
31
32
33
34
35
36
37
38
39
40
41
42
43
44
45
46
47
48
49
50
2
1
bits
0
Motif 2
1
2
3
4
5
6
7
8
9
10
11
12
13
14
15
16
17
18
19
20
21
22
23
24
25
26
27
28
29
30
31
32
33
34
35
36
37
38
39
40
41
42
43
44
45
46
47
48
49
50
2
1
bits
0
Motif 3
1
2
3
4
5
6
7
8
9
10
11
12
13
14
15
16
17
18
19
20
21
22
23
24
25
26
27
28
29
30
31
32
33
34
35
36
37
38
39
40
41
42
43
44
45
46
47
48
49
50
2
1
bits
0
Motif 4
1
2
3
4
5
6
7
8
9
10
11
12
13
14
15
16
17
18
19
20
21
22
23
24
25
26
27
28
29
30
31
32
33
34
35
36
37
38
39
40
41
42
43
44
45
46
47
48
49
50
2
1
bits
0
Motif 5
1
2
3
4
5
6
7
8
9
10
11
12
13
14
15
16
17
18
19
20
21
22
23
24
25
26
27
28
29
30
31
32
33
34
35
36
37
38
39
40
41
42
43
44
45
46
47
48
49
50
2
1
bits
0
Motif 6
1
2
3
4
5
6
7
8
9
10
11
12
13
14
15
16
17
18
19
20
21
22
23
24
25
26
27
28
29
30
31
32
33
34
35
36
37
38
39
40
41
42
43
44
45
46
47
48
49
50
2
1
bits
0
Motif 7
1
2
3
4
5
6
7
8
9
10
11
12
13
14
15
16
17
18
19
20
21
22
23
24
25
26
27
28
29
30
31
32
33
34
35
36
37
38
39
40
41
42
43
44
45
46
47
48
49
50
2
1
bits
0
Motif 8
1
2
3
4
5
6
7
8
9
10
11
12
13
14
15
16
17
18
19
20
21
22
23
24
25
26
27
28
29
30
31
32
33
34
35
36
37
38
39
40
41
42
43
44
45
46
47
48
49
50
2
1
bits
0
Motif 9
1
2
3
4
5
6
7
8
9
10
11
12
13
14
15
16
17
18
19
20
21
22
23
24
25
26
27
28
29
30
31
32
33
34
35
36
37
38
39
40
41
42
43
44
45
46
47
48
49
50
2
1
bits
0
Motif 10
1
2
3
4
5
6
7
8
9
10
11
12
13
14
15
16
17
18
19
20
21
22
23
24
25
26
27
28
29
30
31
32
33
34
35
36
37
38
39
40
41
42
43
44
45
46
47
48
49
50
Figure S1. The conserved motifs of the genes in the PgHDZ gene family determined by MEME. The height of each letter represents the conservation level of the nucleotide across the PgHDZ genes.
