# Supplementary material for: Genome-wide identification and systematic analysis of the HD-Zip gene family and its roles in response to pH in Panax ginseng Meyer
Source: BMC Plant Biol. 2023 Jan 13;23:30. doi: 10.1186/s12870-023-04038-9 (PMC9838044; doi:10.1186/s12870-023-04038-9)
Supplement: Supplementary file 2 — Additional file 2: Fig. S2. Expressions of the 117 PgHDZ gene transcripts in four-year-old plant roots of 42 cultivars. (a) Percentage of PgHDZ transcripts expressed in 42 cultivars with 117 transcripts randomly selected from the ginseng transcriptome as the reference. (b) Expression heatmap of the PgHDZ transcripts in different cultivars. [file 12870_2023_4038_MOESM2_ESM.pptx]

## Slide 1
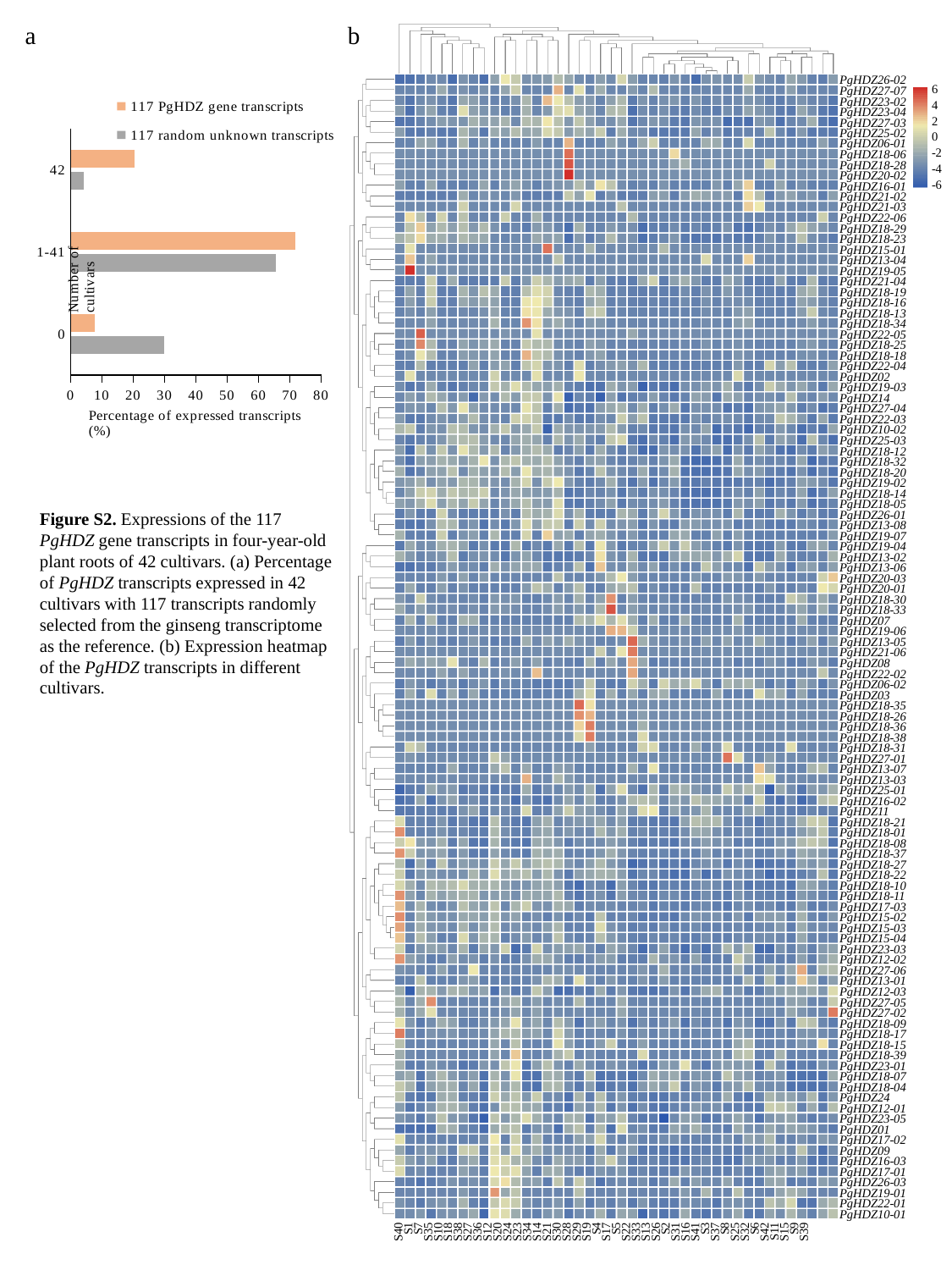

a
b
### Chart
| Category | 117 random unknown transcripts | 117 PgHDZ gene transcripts |
|---|---|---|
| 0 | 29.91 | 7.69 |
| 1-41 | 65.50700000000002 | 71.73000000000003 |
| 42 | 4.27 | 20.51 |PgHDZ26-02
PgHDZ27-07
PgHDZ23-02
PgHDZ23-04
PgHDZ27-03
PgHDZ25-02
PgHDZ06-01
PgHDZ18-06
PgHDZ18-28
PgHDZ20-02
PgHDZ16-01
PgHDZ21-02
PgHDZ21-03
PgHDZ22-06
PgHDZ18-29
PgHDZ18-23
PgHDZ15-01
PgHDZ13-04
PgHDZ19-05
PgHDZ21-04
PgHDZ18-19
PgHDZ18-16
PgHDZ18-13
PgHDZ18-34
PgHDZ22-05
PgHDZ18-25
PgHDZ18-18
PgHDZ22-04
PgHDZ02
PgHDZ19-03
PgHDZ14
PgHDZ27-04
PgHDZ22-03
PgHDZ10-02
PgHDZ25-03
PgHDZ18-12
PgHDZ18-32
PgHDZ18-20
PgHDZ19-02
PgHDZ18-14
PgHDZ18-05
PgHDZ26-01
PgHDZ13-08
PgHDZ19-07
PgHDZ19-04
PgHDZ13-02
PgHDZ13-06
PgHDZ20-03
PgHDZ20-01
PgHDZ18-30
PgHDZ18-33
PgHDZ07
PgHDZ19-06
PgHDZ13-05
PgHDZ21-06
PgHDZ08
PgHDZ22-02
PgHDZ06-02
PgHDZ03
PgHDZ18-35
PgHDZ18-26
PgHDZ18-36
PgHDZ18-38
PgHDZ18-31
PgHDZ27-01
PgHDZ13-07
PgHDZ13-03
PgHDZ25-01
PgHDZ16-02
PgHDZ11
PgHDZ18-21
PgHDZ18-01
PgHDZ18-08
PgHDZ18-37
PgHDZ18-27
PgHDZ18-22
PgHDZ18-10
PgHDZ18-11
PgHDZ17-03
PgHDZ15-02
PgHDZ15-03
PgHDZ15-04
PgHDZ23-03
PgHDZ12-02
PgHDZ27-06
PgHDZ13-01
PgHDZ12-03
PgHDZ27-05
PgHDZ27-02
PgHDZ18-09
PgHDZ18-17
PgHDZ18-15
PgHDZ18-39
PgHDZ23-01
PgHDZ18-07
PgHDZ18-04
PgHDZ24
PgHDZ12-01
PgHDZ23-05
PgHDZ01
PgHDZ17-02
PgHDZ09
PgHDZ16-03
PgHDZ17-01
PgHDZ26-03
PgHDZ19-01
PgHDZ22-01
PgHDZ10-01
6
4
2
0
-2
-4
-6
Figure S2. Expressions of the 117 PgHDZ gene transcripts in four-year-old plant roots of 42 cultivars. (a) Percentage of PgHDZ transcripts expressed in 42 cultivars with 117 transcripts randomly selected from the ginseng transcriptome as the reference. (b) Expression heatmap of the PgHDZ transcripts in different cultivars.
S40
S1
S7
S35
S10
S18
S38
S27
S36
S12
S20
S24
S23
S34
S14
S21
S30
S28
S29
S19
S4
S17
S5
S22
S33
S13
S26
S2
S31
S16
S41
S3
S37
S8
S25
S32
S6
S42
S11
S15
S9
S39
