# Supplementary material for: Genome-wide identification and systematic analysis of the HD-Zip gene family and its roles in response to pH in Panax ginseng Meyer
Source: BMC Plant Biol. 2023 Jan 13;23:30. doi: 10.1186/s12870-023-04038-9 (PMC9838044; doi:10.1186/s12870-023-04038-9)
Supplement: Supplementary file 3 — Additional file 3: Fig. S3. The average expression of the 19 PgHDZ transcripts expressed in all 42 cultivars, 14 tissues and four aged roots. The genes belonging to different subfamilies were shown, with the genes black font from subfamily I, the genes in pink font from subfamily II, and the genes in blue font from subfamily III. [file 12870_2023_4038_MOESM3_ESM.pptx]

## Slide 1
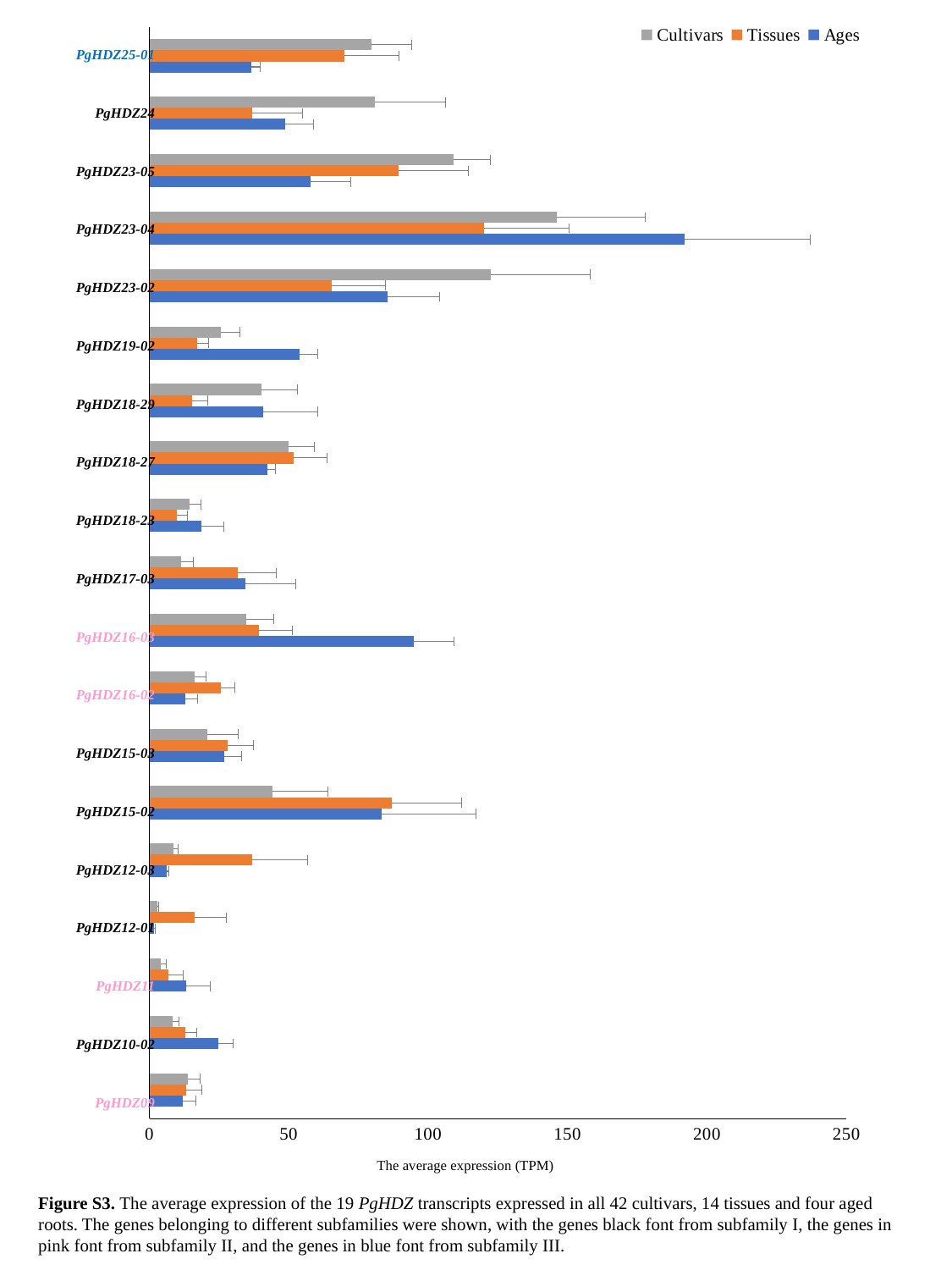

### Chart
| Category | Ages | Tissues | Cultivars |
|---|---|---|---|
| PgHDZ09 | 12.025 | 13.21928571 | 13.62785714 |
| PgHDZ10-02 | 24.7575 | 12.73571429 | 8.406190476 |
| PgHDZ11 | 13.055 | 6.909285714 | 4.171428571 |
| PgHDZ12-01 | 1.675 | 16.28428571 | 2.710952381 |
| PgHDZ12-03 | 6.095 | 36.99642857 | 8.638571429 |
| PgHDZ15-02 | 83.3775 | 87.07285714 | 44.25785714 |
| PgHDZ15-03 | 26.7075 | 28.01928571 | 20.6497619 |
| PgHDZ16-02 | 12.9825 | 25.74428571 | 16.33619048 |
| PgHDZ16-03 | 94.8725 | 39.40357143 | 34.6547619 |
| PgHDZ17-03 | 34.335 | 31.59214286 | 11.43071429 |
| PgHDZ18-23 | 18.7525 | 9.929285714 | 14.25642857 |
| PgHDZ18-27 | 42.4025 | 51.775 | 49.75547619 |
| PgHDZ18-29 | 40.675 | 15.20642857 | 40.12833333 |
| PgHDZ19-02 | 53.8875 | 17.11071429 | 25.64333333 |
| PgHDZ23-02 | 85.475 | 65.42142857 | 122.4778571 |
| PgHDZ23-04 | 191.8275 | 119.99 | 146.2047619 |
| PgHDZ23-05 | 57.92 | 89.28857143 | 109.0490476 |
| PgHDZ24 | 48.8175 | 36.71857143 | 80.91761905 |
| PgHDZ25-01 | 36.62 | 70.015 | 79.67547619 |PgHDZ25-01
PgHDZ24
PgHDZ23-05
PgHDZ23-04
PgHDZ23-02
PgHDZ19-02
PgHDZ18-29
PgHDZ18-27
PgHDZ18-23
PgHDZ17-03
PgHDZ16-03
PgHDZ16-02
PgHDZ15-03
PgHDZ15-02
PgHDZ12-03
PgHDZ12-01
PgHDZ11
PgHDZ10-02
PgHDZ09
The average expression (TPM)
Figure S3. The average expression of the 19 PgHDZ transcripts expressed in all 42 cultivars, 14 tissues and four aged roots. The genes belonging to different subfamilies were shown, with the genes black font from subfamily I, the genes in pink font from subfamily II, and the genes in blue font from subfamily III.
