# Supplementary material for: Genome-wide identification and systematic analysis of the HD-Zip gene family and its roles in response to pH in Panax ginseng Meyer
Source: BMC Plant Biol. 2023 Jan 13;23:30. doi: 10.1186/s12870-023-04038-9 (PMC9838044; doi:10.1186/s12870-023-04038-9)
Supplement: Supplementary file 4 — Additional file 4: Fig. S4. The co-expression network of the PgHDZ gene transcripts expressed in four-year-old plant roots of 42 cultivars. (a) The co-expression network of the PgHDZ gene transcripts constructed at p ≤ 5.0E-02. (b) Ten clusters of the co-expression network. (c and d) The co-expression network formation tendency of the PgHDZ gene transcripts presented by the number of nodes (c) and the number of edges (d). (e and f) Statistics of the co-expression network formation tendency of the PgHDZ gene transcripts presented by the number of nodes (e) and the number of edges (f). The 100 PgHDZ and unknown transcripts were randomly selected with 20 bootstraps. The capital letter represents that the difference between PgHDZ and random unknown transcripts was significant at p ≤ 1.0E-02. [file 12870_2023_4038_MOESM4_ESM.pptx]

## Slide 1
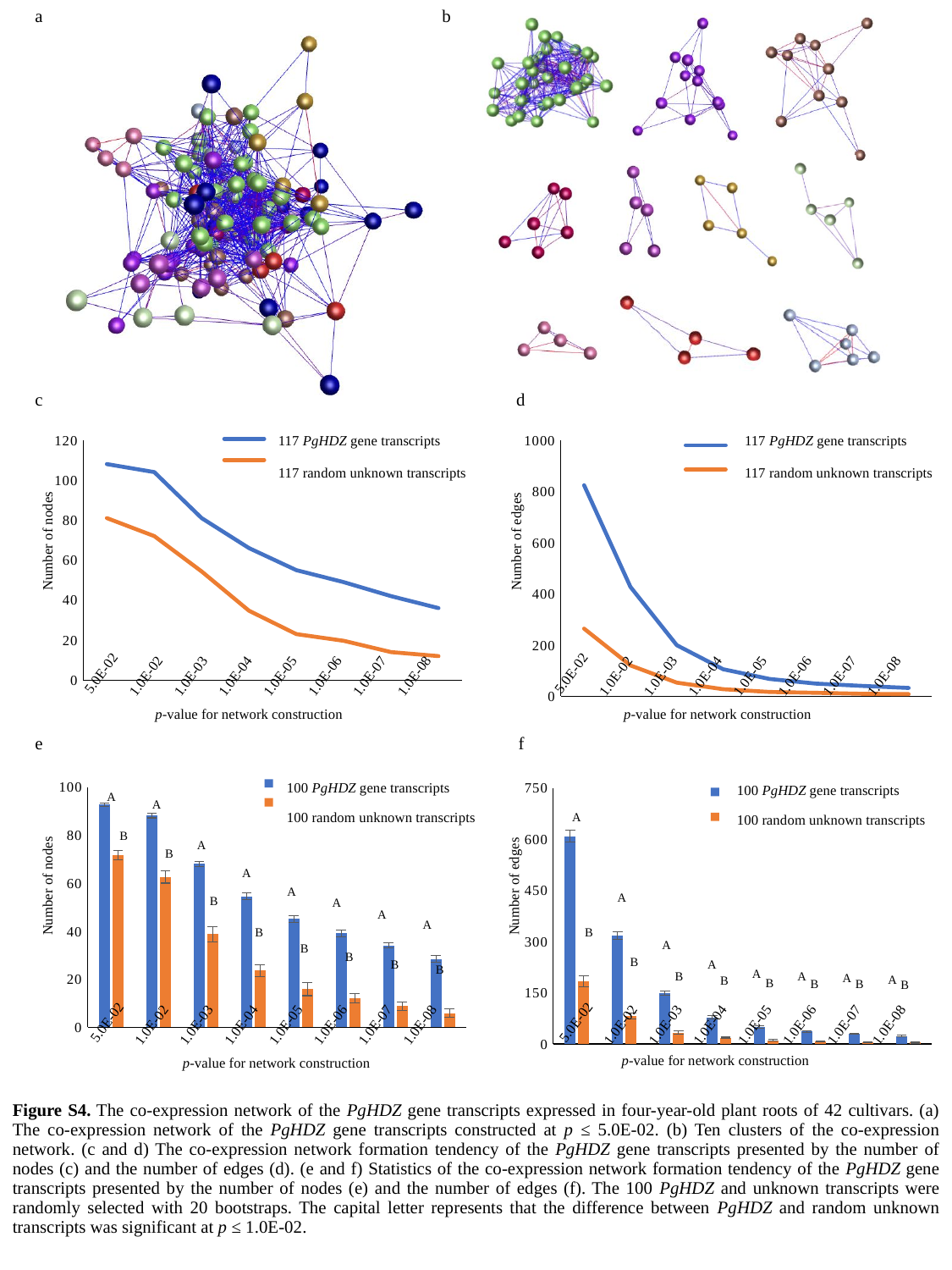

a
b
c
d
### Chart
| Category | 117 PgHD-Zip gene transcripts | 117 random unknown transcritps |
|---|---|---|
| 0.05 | 108.0 | 81.0 |
| 0.01 | 104.0 | 72.0 |
| 1E-3 | 81.0 | 54.333333333333336 |
| 1E-4 | 66.0 | 34.666666666666664 |
| 1.0000000000000001E-5 | 55.0 | 23.0 |
| 9.9999999999999995E-7 | 49.0 | 19.666666666666668 |
| 9.9999999999999995E-8 | 42.0 | 14.0 |
| 1E-8 | 36.0 | 12.0 |117 PgHDZ gene transcripts
117 random unknown transcripts
5.0E-02
1.0E-02
1.0E-03
1.0E-04
1.0E-05
1.0E-06
1.0E-07
1.0E-08
p-value for network construction
### Chart
| Category | 117 PgHD-Zip gene transcripts | 117 random unknown transcritps |
|---|---|---|
| 0.05 | 824.0 | 263.6666666666667 |
| 0.01 | 427.0 | 120.0 |
| 1E-3 | 199.0 | 52.666666666666664 |
| 1E-4 | 105.0 | 27.0 |
| 1.0000000000000001E-5 | 67.0 | 16.333333333333332 |
| 9.9999999999999995E-7 | 49.0 | 13.0 |
| 9.9999999999999995E-8 | 40.0 | 9.0 |
| 1E-8 | 32.0 | 8.0 |117 PgHDZ gene transcripts
117 random unknown transcripts
5.0E-02
1.0E-02
1.0E-03
1.0E-04
1.0E-05
1.0E-06
1.0E-07
1.0E-08
p-value for network construction
Number of nodes
Number of edges
e
f
### Chart
| Category | 100 PgHD-Zip gene transcripts | 100 random unknown transcripts |
|---|---|---|
| 0.05 | 92.65 | 71.65 |
| 0.01 | 88.15 | 62.55 |
| 1E-3 | 68.05 | 38.7 |
| 1E-4 | 54.45 | 23.6 |
| 1.0000000000000001E-5 | 45.15 | 15.75 |
| 9.9999999999999995E-7 | 39.1 | 11.9 |
| 9.9999999999999995E-8 | 34.05 | 8.7 |
| 1E-8 | 28.4 | 5.85 |A
A
B
A
B
A
A
B
A
A
A
B
B
B
B
B
100 PgHDZ gene transcripts
100 random unknown transcripts
Number of nodes
p-value for network construction
5.0E-02
1.0E-02
1.0E-03
1.0E-04
1.0E-05
1.0E-06
1.0E-07
1.0E-08
### Chart
| Category | 100 PgHD-Zip gene transcripts | 100 random unknown transcripts |
|---|---|---|
| 0.05 | 609.0 | 183.55 |
| 0.01 | 318.45 | 81.3 |
| 1E-3 | 148.45 | 33.2 |
| 1E-4 | 77.75 | 17.75 |
| 1.0000000000000001E-5 | 49.8 | 10.9 |
| 9.9999999999999995E-7 | 36.45 | 7.75 |
| 9.9999999999999995E-8 | 29.8 | 5.7 |
| 1E-8 | 23.5 | 3.7 |100 PgHDZ gene transcripts
A
100 random unknown transcripts
Number of edges
A
B
A
B
A
A
B
A
A
A
B
B
B
B
B
p-value for network construction
5.0E-02
1.0E-02
1.0E-03
1.0E-04
1.0E-05
1.0E-06
1.0E-07
1.0E-08
Figure S4. The co-expression network of the PgHDZ gene transcripts expressed in four-year-old plant roots of 42 cultivars. (a) The co-expression network of the PgHDZ gene transcripts constructed at p ≤ 5.0E-02. (b) Ten clusters of the co-expression network. (c and d) The co-expression network formation tendency of the PgHDZ gene transcripts presented by the number of nodes (c) and the number of edges (d). (e and f) Statistics of the co-expression network formation tendency of the PgHDZ gene transcripts presented by the number of nodes (e) and the number of edges (f). The 100 PgHDZ and unknown transcripts were randomly selected with 20 bootstraps. The capital letter represents that the difference between PgHDZ and random unknown transcripts was significant at p ≤ 1.0E-02.
